# The Oral Bacterial Community in *Melanophryniscus admirabilis* (Admirable Red-Belly Toads): Implications for Conservation

**DOI:** 10.3390/microorganisms9020220

**Published:** 2021-01-22

**Authors:** Michele Bertoni Mann, Janira Prichula, Ícaro Maia Santos de Castro, Juliana Mello Severo, Michelle Abadie, Thayná Mendes De Freitas Lima, Valentina Caorsi, Márcio Borges-Martins, Jeverson Frazzon, Ana Paula Guedes Frazzon

**Affiliations:** 1 Post-Graduation Program in Agricole and Environmental Microbiology, Department of Microbiology, Immunology and Parasitology, Federal University of Rio Grande do Sul, Porto Alegre 90050-170, Brazil; mbertonimann@gmail.com; 2 Department of Health Sciences, Federal University of Health Sciences of Porto Alegre, Porto Alegre 90050-170, Brazil; janira.pri@gmail.com (J.P.); icaromscastro@gmail.com (Í.M.S.d.C.); juliana.bioinformatica@gmail.com (J.M.S.); 3 Biosciences Institute, Federal University of Rio Grande do Sul, Porto Alegre 91509-900, Brazil; abadie.mi@gmail.com; 4 Post-Graduation Program in Animal Biology, Department of Zoology, Biosciences Institute, Federal University of Rio Grande do Sul, Porto Alegre 91509-900, Brazil; thaynamfl@gmail.com (T.M.D.F.L.); valenzc@gmail.com (V.C.); borges.martins@ufrgs.br (M.B.-M.); 5 Research and Innovation Centre, The Institute Agrario di San Michele all’Adige, Fondazione Edmund Mach, 1 38010 San Michele all’Adige, Italy; 6 Biochemistry and Molecular Biology of Microorganisms Laboratory, Federal University of Rio Grande do Sul, Porto Alegre 91509-900, Brazil; jeverson.frazzon@ufrgs.br

**Keywords:** high-throughput sequencing, amphibian, bacteria, xenobiotic, anthropogenic action

## Abstract

*Melanophryniscus admirabilis* (admirable red-belly toad) is a microendemic and critically endangered species found exclusively along 700 m of the Forqueta River, in a fragment of the Atlantic Forest of southern Brazil. One of the greatest concerns regarding the conservation of this species is the extensive use of pesticides in areas surrounding their natural habitat. In recent years, the adaptation and persistence of animal species in human-impacted environments have been associated with microbiota. Therefore, the present study aimed to characterize the oral bacterial community of wild *M. admirabilis* and to address the question of how this community might contribute to this toad’s adaptation in the anthropogenic environment as well as its general metabolic capabilities. A total of 11 oral samples collected from wild *M. admirabilis* were characterized and analyzed via high-throughput sequencing. Fragments of the *16S rRNA* variable region 4 (V4) were amplified, and sequencing was conducted using an Ion Personal Genome Machine (PGM) System with 316 chips. A total of 181,350 sequences were obtained, resulting in 16 phyla, 34 classes, 39 orders, and 77 families. Proteobacteria dominated (53%) the oral microbiota of toads, followed by Firmicutes (18%), Bacteroidetes (17%), and Actinobacteria (5%). No significant differences in microbial community profile from among the samples were reported, which suggests that the low dietary diversity observed in this population may directly influence the bacterial composition. Inferences of microbiome function were performed using PICRUSt2 software. Important pathways (e.g., xenobiotic degradation pathways for pesticides and aromatic phenolic compounds) were detected, which suggests that the bacterial communities may serve important roles in *M. admirabilis* health and survival in the anthropogenic environment. Overall, our results have important implications for the conservation and management of this microendemic and critically endangered species.

## 1. Introduction

Amphibians are highly sensitive to environmental changes and represent the most threatened vertebrate group, with approximately 33% (2390) of the 7166 known species listed in a threatened category [1,2,3]. The anuran genus *Melanophryniscus* (Bufonidae) comprises 29 species of small toads that are geographically restricted to South America, occurring in Brazil, Paraguay, Bolivia, Uruguay, and Argentina [4]. Many species have restricted distributions and are globally listed as threatened, near threatened [5], or data deficient [3]. Among the 22 species of *Melanophryniscus* found in Brazil, *Melanophryniscus admirabilis* (admirable red-belly toad) is a microendemic species found exclusively along 700 m of the Forqueta River in a fragment of the Atlantic Forest of southern Brazil [6]. *M. admirabilis* is one of the largest species in the genus (females grow up to 40 mm, while males are smaller) [4]. They are easily distinguishable by their green dorsum with a black belly as well as contrasting yellowish glands and red palms, soles, and inguinal region [6]. Its conspicuous coloration—associated with unken reflex behavior—is supposedly a warning signal for potential predators that indicates toxicity due to their skin`s alkaloid compounds. These alkaloids are sequestered from their arthropod-rich diet (e.g., ants, beetles, mites, and millipedes) and is released through their multicellular exocrine glands [7]. Approximately 170 alkaloids and 15 structural classes have already been identified in 9 species of *Melanophryniscus* [8], with 5,8-disubstituted indolizidines, 5,6,8-trisubstituted indolizidines, pumiliotoxins, tricyclics, and decahydroquinolines being the most commonly observed [9,10,11].

*Melanophryniscus admirabilis* is officially listed by International Union for Conservation of Nature (IUCN) as critically endangered and is part of the Action Plan for the Conservation of Amphibians and Reptiles in southern Brazil [6]. Notably, this species’ main threats include the ongoing loss of habitat quality resulting from anthropogenic infrastructures and activities such as hydroelectric power generation, deforestation, pesticide use in tobacco and soybean plantations, livestock activity, the illegal pet trade, and trampling by tourists at reproductive sites [3]. Due to the vulnerability of the only known population of this species, the conservation of *M. admirabilis* is a priority in Brazil. The few studies involving this species largely focused on its biology, ecology [12], and ecotoxicology [13]. To date, no work has been carried out to examine the microbiota composition and/or diversity in this species. Notably, microbes serve an important role in maintaining animal health. Additionally, the adaptation and persistence of animal species in human-impacted environments have been associated with microbiota [2,14]. Determining microbiota composition could promote a greater understanding of species’ physiological statuses and niche divergences under differing environmental conditions [15,16].

To date, amphibian skin and gut microbiomes have been relatively well studied; however, studies involving oral microbiome remain scarce [2,17,18,19]. The composition, diversity, and function of microbial communities mirrors host species’ health maintenance in the environment and may also reflect the ecological condition of the habitat. The composition of microbiota in frogs is governed by the endogenous environment, shaped by their physical, physiological, and immune properties, and influenced by their surrounding environments via their diet, which constitutes an important source of organisms in the oral and gastrointestinal tracts of these animals. To ensure the successful conservation of *M. admirabilis*, it is important to assess the microbiota of this species. Therefore, the present study aimed to characterize the oral bacterial community of wild *M. admirabilis* and address the question of how this community might contribute to this species’ adaptation in the anthropogenic environment as well as its general metabolic capabilities.

## 2. Materials and Methods

### 2.1. Sample Collection

**Figure 1:**
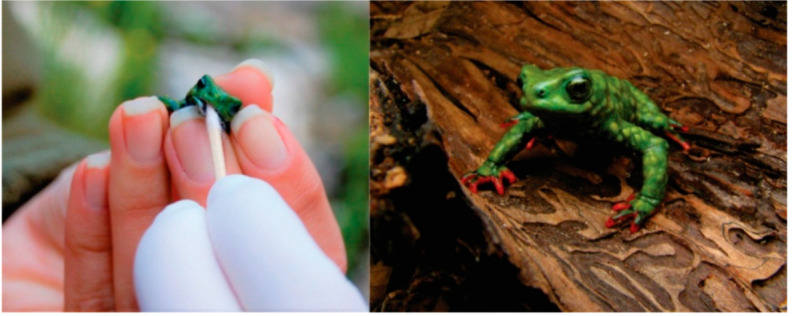
Wild *Melanophryniscus admirabilis* (admirable red-belly toads) in the Forqueta river’s margins at the Perau de Janeiro Arvorezinha, South Brazil. Oral sample collected from the *M. admirabilis* (**left**; Photo: Márcio Borges-Martins). *Melanophryniscus admirabilis* in the breeding sites in the Forqueta river’s margins at the Perau de Janeiro (**right**; Photo: Márcio Borges-Martins).

Eleven oral samples were collected from wild *Melanophryniscus admirabilis* (Figure 1; Table 1). Samples were taken from active toads in the breeding sites in the Forqueta river’s margins in the Perau de Janeiro locality, Arvorezinha, Rio Grande do Sul, Brazil (52°18′ W, 28°51′ S). The area is situated at the southern end of the Atlantic Forest, in a transitional phytoecological region between the mixed ombrophilous forest and the deciduous seasonal forest [20].

The oral swab collection was performed according to the sample collection protocol [21]. Oral samples were collected using commercially available sterile cotton-tipped swab sticks. All samples were placed in sterile tubes, kept on ice, and sent to our laboratory for storage at −80 °C. The toads were released back into the wild immediately after the sample collection. All specimens were individually marked using a photo identification protocol [12].

### 2.2. Ethics and Sampling Permits

This study was carried out following the recommendations of the Chico Mendes Institute for Biodiversity Conservation (ICMBio) and was approved by the Research and Ethics Committees at the Federal University of Rio Grande do Sul (Projects 19541, 25526, and 25528). The protocol was approved by the Information and Authorization System in Biodiversity (SISBIO), numbers 40004-5 and 10341-1 (for M. Borges-Martins). All possible measures were taken to reduce the impact of our sampling protocol, which is part of a larger program intended for the study, monitoring, and conservation of the only known admirable red-belly toad population. Research priorities and protocols are also part of the Action Plan for the Conservation of Amphibians and Reptiles in southern Brazil [12].

### 2.3. DNA Extraction, PCR-Amplification of Bacterial 16S rRNA Genes and Sequencing

Total DNA from the oral swab samples was extracted using a DNeasy Blood and Tissue Kit (Qiagen, Valencia, CA, USA), according to the manufacturer’s instructions. The DNA concentration was determined using the Qubit, and DNA quality was verified using the NanoDrop ND-1000 (Thermo Fisher Scientific, Waltham, MA, USA).

To characterize the bacterial community present in each oral sample, fragments of the *16S rRNA* gene were amplified using the primers 515F and 806R [22]. Multiple samples were PCR-amplified using barcoded primers linked with the Ion adapter “A” sequence and the Ion adapter “P1′′ sequence to obtain a sequence of primer composed for A-barcode-806R and P1-515F adapter and primers. PCR reactions were carried out with the Platinum *Taq* DNA Polymerase High Fidelity kit (Invitrogen, Carlsbad, CA, USA). PCR was performed with High Fidelity PCR buffer, 2U of Platinum *Taq* DNA Polymerase, 2 mM of MgSO_4_, 0.2 mM of dNTP Mix, 25 μg of Ultrapure BSA (Invitrogen, Carlsbad, CA, USA), 0.1 μM of each forward primer, approximately 30 ng of DNA template, and ultrapure water to complete a final volume of 25 μL per reaction. The PCR conditions were 94 °C for 5 min, followed by 30 cycles of 94 °C for 45 s, 56 °C for 45 s, and 68 °C for 1 min, and a final extension step of 68 °C for 10 min.

Samples were sequenced at the Universidade Federal do Pampa (UNIPAMPA, Bagé, RS, Brazil). After purifying PCR amplicons using Agencount AMPure Beads (Beckman Coulter), library preparation was carried out with the Ion OneTouch^TM^ 2 System fitted with the Ion PGMTM OT2 400 Kit Template (Thermo Fisher Scientific, Waltham, MA, USA) from an initial amount of 100 ng of PCR product. Because all samples were sequenced in a multiplexed Personal Genome Machine (PGM) run, barcode sequences were used to identify each sample from the total sequencing output. Sequencing was conducted on an Ion Personal Genome Machine (PGM) System (Thermo Fisher Scientific, Waltham, MA, USA) with 316 chips, following the manufacturer’s instructions. Sequences have been submitted to the European Molecular Biology Laboratory (EMBL) database under accession number PRJEB33232. Despite the short-read lengths (~290 bp), this targeted gene region should also provide sufficient resolution.

### 2.4. Bacterial Community Analysis

Bioinformatics analysis of *16S rRNA* amplicons was performed using QIIME 2 version 2019.7 [22]. Raw sequence data were quality filtered, denoised, and chimera filtered using the q2-dada2- plugin with DADA2 pipeline Callahan [23]. The 5′ and 3′ nucleotide bases were trimmed from forward and reverse read sequences due to low quality. Reads with several expected errors higher than 4 were discarded. Read length filtering was applied, and the reads were trimmed at the first instance of a quality score less than or equal to 2. The resulting reads were truncated at 200 bp length. Chimera removal was performed using the consensus method. The amplicon sequence variants (ASVs) obtained by DADA2 pipeline were merged into a single feature table using the q2-feature-table plugin.

The ASV’s were aligned with multiple alignment using fast Fourier transform (MAFFT) (via q2-alignment) [24]. Taxonomy was assigned to the classify-sklearn naive Bayes taxonomy classifier [25]. The classifier was trained using extracted Greengenes 13_8 reference sequences with 99% similarity from *16S rRNA* variable region 4 (V4). The resulting feature table, rooted tree from reconstructed phylogeny, and taxonomy classification were imported from QIIME2 to the R v3.6.1 environment for further data analysis using Microbiome v1.6.0 and Phyloseq v1.28.0 [26,27]. For Taxonomic analysis, the feature table was transformed to compositional data for taxa bar plot composition visualization of the five most abundant phylum and families using the plot composition function from Microbiome R package [27]. 

The taxon diversity study (richness and evenness) within the samples was performed by employing the Shannon diversity, the InvSimpson diversity, and the Chao1 index, whereas the observed species metrics calculation and diversity between samples were estimated using Microbiome and Phyloseq packages in R. The significance was estimated with a pairwise comparison using a non-parametric test Wilcoxon [28], using functions from the Microbiome R package.

### 2.5. Functional Predictions from Amplicon Sequences

A predictive functional profile of the oral bacterial community was conducted using PICRUSt2 software [29] against Kyoto Encyclopedia of Genes and Genomes (KEGG) database [30]. PICRUSt2 output is a biom table with rows in terms of functional orthologs (KO) and samples as columns. The KO terms levels were mapped into KEGG levels and imported to statistical analysis of taxonomic and functional profiles (STAMP) software for statistical analysis [31]. Briefly, samples were divided into two gender groups (F-M), and a Welch’s *t*-test was performed to evaluate the significance of functional predictions with *p*-value < 0.05. Benjamini–Hochberg adjusted *p*-value was calculated to control the false discovery rate (FDR) in multiple testing. The KEGG groups were considered significantly enriched by satisfying an FDR corrected *p*-value of 0.05.

## 3. Results

A total of 181,350 sequences were obtained from the oral samples of wild *Melanophryniscus admirabilis* after discarding substandard sequences. Among these cleaned sequences, we obtained 13,650 ASVs per sample, which were grouped into 1039 (ASVs). Sequence analysis grouped the reads into 16 phyla, 34 classes, 39 orders, and 77 families.

**Figure 2:**
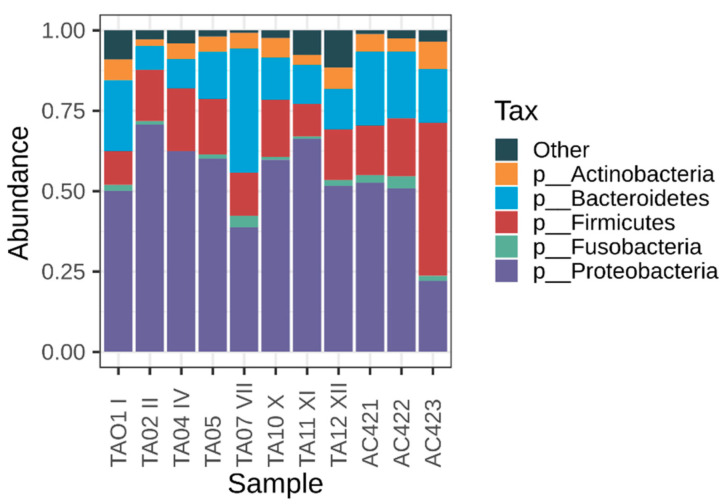
Oral bacterial composition of wild *Melanophryniscus admirabilis* (admirable red-belly toads). Taxonomic composition of the oral microbiota among the eleven samples was compared based on the relative abundance (reads of a taxon/total reads in a sample).

Five phyla presented relative abundances greater than 1% and were present in all evaluated samples. Among phyla, Proteobacteria dominated the oral microbiota of wild *M. admirabilis*, with the highest relative abundance (53%), followed by Firmicutes (18%), Bacteroidetes (17%), Actinobacteria (5%), and Fusobacteria (2%) (Figure 2; Table S1). These sequences belonged mainly to seven orders: Burkholderiales (23%), Bacteroidales (14%), Lactobacillales (8%), Clostridiales (8%), Enterobacteriales (7%) Pseudomonadales (5%), and Actinomycetales (5%) (Table S2).

While 77 families were detected in the oral samples, only 28 families exhibited a relative average abundance of ≥1% (Table S3). Burkholderiaceae (16%), Prevotellaceae (10%), Enterobacteriaceae (7%), Comamonadaceae (6%), and Streptococcaceae (6%) were more abundant and were present in all samples evaluated (Figure S1).

**Figure 3:**
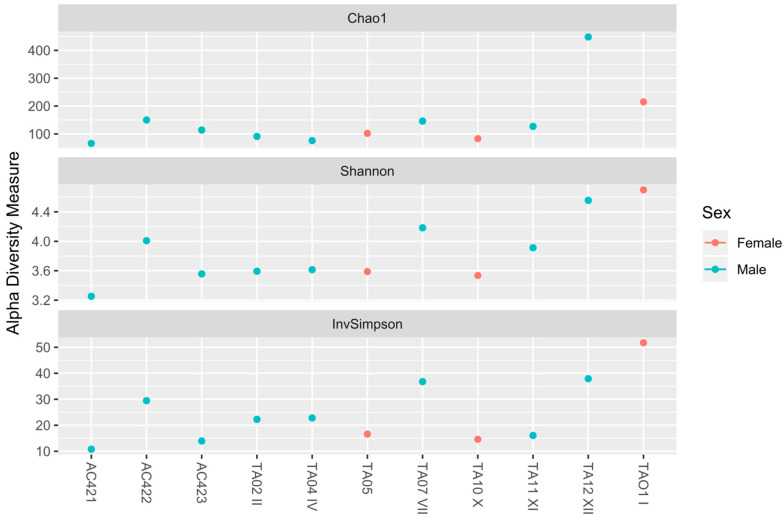
Alpha diversity comparisons of oral bacterial microbiota of wild *Melanophryniscus admirabilis* (admirable red-belly toads). Alpha-diversity analysis based on Chao 1 diversity (**top**), Shannon diversity (**middle**), and InvSimpson diversity (**bottom**), measure of species richness based on amplicon sequence variants (ASVs) of the eleven oral samples collected from wild *M. admirabilis*. No significant difference among the samples was observed.

Alpha diversity metrics (Shannon, Chao1, Inverse Simpson index) did not exhibit any identifiable change (*p*-value > 0.05) in bacterial community structure grouping samples by sex (Figure 3).

PICRUSt2 software was used to better understand the important role of the oral bacterial microbiota present in wild *M. admirabilis*. Metabolic functions were enriched in our samples, and functional features in 26 pathways were observed, including membrane transport proteins, amino acids metabolism, carbohydrate metabolism, energy metabolism, replication and repair systems, cofactor and vitamin metabolism, nucleotide metabolism, xenobiotic biodegradation metabolism, lipid metabolism, the metabolism of other amino acids, polypeptide and terpenoid metabolism, and the biosynthesis of other secondary metabolites (Figure S2).

**Figure 4:**
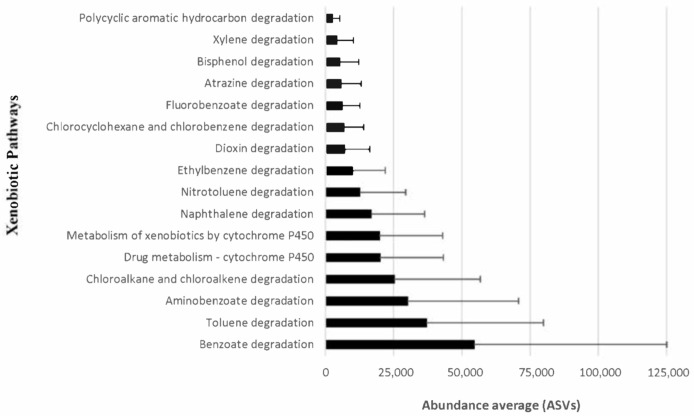
Average number of amplicon sequence variants (ASVs) >1% average relative abundance) among the *16S rRNA* gene profiles of oral samples from wild *Melanophryniscus admirabilis* belonging to the predicted xenobiotic pathways in relative Kyoto Encyclopedia of Genes and Genomes (KEEG) Level 2.

We correlated the microbial functional features (e.g., xenobiotic degradation and metabolism) with toad habitat and diet. A total of 16 pathways were identified using PICRUSt2 software (Figure 4; Table S4). Two of these pathways were related to benzoate and toluene degradation, with an elevated frequency of amplicon sequence variants (average number of ASVs = 54,486 and 36,950, respectively). The data analysis showed a higher standard deviation for benzoate degradation and toluene degradation when compared to polycyclic aromatic hydrocarbon degradation (Table S4). Other groups of xenobiotic activity included aminobenzoate degradation (average number of ASVs = 30,067), chloroalkane and chloroalkene degradation (average number of ASVs = 25,160), drug metabolism-cytochrome P450 (average number of ASVs = 19,923), naphthalene degradation (average number of ASVs = 19,789), nitrotoluene degradation (average number of ASVs = 12,598), ethylbenzene degradation (average number of ASVs = 9718), dioxin degradation and biosynthesis (average number of ASVs = 6847), atrazine degradation (average number of ASVs = 5576), and fluorobenzoate degradation (average number of ASVs = 5864) (Table S4).

## 4. Discussion

The number of amphibian microbiome studies has been increasing in recent years to facilitate an improved understanding of the diverse communities of bacteria, fungi, and viruses that inhabit their bodies [2,17,18,19,32,33]. The skin microbiome has been extensively studied due to its relationship to an emergent disease caused by the chytrid fungus (*Batrachochytrium dendrobatidis*) [2,32]. However, knowledge regarding the taxonomic content of amphibian oral microbiota remains extremely limited [2,17,18,19]. In the present study, we describe the bacterial communities present in the oral cavity of wild *Melanophryniscus admirabilis* with the use of high-throughput sequencing for the first time

Proteobacteria, Firmicutes, Bacteroidetes, Actinobacteria, and Fusobacteria (which accounted for 95% of the oral microbial community composition), represent typical mucosal taxa and were shared among all samples. These microbial phyla have been associated with symbiotic roles and are commonly observed in the amphibian gastrointestinal tract [34,35,36]. Chang et al. [33] reported that Bacteroides, Firmicutes, and Proteobacteria were also dominant among the intestinal microbiota of rice frogs (*Fejervarya limnocharis*) in natural and farmland habitats. Moreover, according to a study performed in Canada and the United States, over 75% of the gut microbial composition of *Rana pipiens* (northern leopard frogs) included Proteobacteria and Firmicutes [37].

In oral samples of wild *M. admirabilis,* Proteobacteria represented 53% of the assigned sequence variants. Notably, the diet route constitutes an important source of organisms in the oral and gastrointestinal tract of these animals. The presence of Proteobacteria in oral samples may be associated with the diet of this species being arthropod-rich because this phylum was observed as dominant in the cuticular microbiomes of ants and the gut microbiomes of arthropods [38]. However, the predominance of this phylum may also be associated with the ability of *M. admirabilis* to synthesize bioactive secondary metabolites that are frequently observed in several bacteria of this phylum [39]. For example, *Janthinobacterium lividum* was present in amphibian guts and inhibited the growth of lethal amphibian fungus [40]. Additionally, antifungal activity from the genus *Pseudomonas* was discovered on the skin of *Rana muscosa* (mountain yellow-legged frogs) and *R. pipiens* [41,42]. In this sense, the oral microbiota of amphibians should be investigated further because it may be a source of compounds with antimicrobial activity that affects their associated microbial community diversity or composition.

The oral cavity of wild *M. admirabilis* was dominated by the orders Burkholderiales (Burkholderiaceae and Comamonadaceae), Enterobacteriales (Enterobacteriaceae), and Bacteroidales (Prevotellaceae). Some microorganisms belonging to these orders coexist with frogs in their habitat. For example, Burkholderiales was found in the skins of terrestrial (*Rhinella marina*, *Litoria nasuta*, and *Limnodynases convexiusculus*) and arboreal (*L. caerulea*, *L. rubella*, and *L. rothii*) anuran species [43]. Furthermore, Burkholderiaceae and Comamonadaceae family members have diverse ecological niches and are found in soil, animals, fungi, and water associated with plants [44,45]. Moreover, Bacteroidales have been reported as symbiotic bacteria essential to the digestive activity of several organisms [40].

In the present study, we observed a similar composition among microbial communities in all oral samples. Recently, Chang et al. [33] hypothesized that the composition of gut microbiota from frogs should be governed by the endogenous gut environment, which is shaped by the physical, physiological, and immune properties of host species, and would be less influenced by the surrounding environment. Here, we suggest that the similarity observed across the oral bacteria communities present in *M. admirabilis* might also be governed by the endogenous oral environment (e.g., saliva [46] and the surrounding environment) because this frog species is microendemic and has a low dietary diversity consisting of arthropods (e.g., Formicidae, Acari, and Coleoptera) that live in this environment [47].

Based on metagenome predictions, one of the most striking observations was xenobiotic degradation in the bacterial community. The communities of bacteria associated with this pathway could suggest positive effects on toad health among populations facing anthropogenic pollutants. *Melanophryniscus admirabilis* belongs to a threatened class of vertebrates, and its observed population decline is due to a combination of different factors, including habitat degradation and fragmentation due to agriculture as well as exposure to contaminants stemming from these activities [12]. Nutrient enrichment from agricultural pollution may reshape the structure of the microbiome composition of aquatic animals and increase their vulnerability to disease [2]. One study analyzed possible alterations to the metabolic and oxidative parameters of total homogenate in *M. admirabilis* tadpoles exposed to two concentrations of commercial formulations containing sulfentrazone (Boral^®^500 SC) and two concentrations containing glyphosate (Roundup^®^ Original). Significant alterations in metabolic and oxidative parameters were observed in groups exposed to sulfentrazone and glyphosate herbicides. However, the tadpoles of this species are capable of moderating potential oxidative lipid damage [13]. Thus, in light of the results of the present study, we suggest that the associated mobilization of enzymes and a microbial community capable of degrading xenobiotics should have a positive effect on the persistence of *M. admirabilis* in this human-impacted environment. Notably, additional factors such as habitat fragmentation, UV radiation, and exposure to other pollutants have negative effects that can interact to affect the survival of these animals. Environmental contaminants such as xenobiotics can alter host-associated microbial communities through the displacement of native bacterial taxa by those capable of withstanding chronic exposure to toxic compounds [48]. Ultimately, improving our knowledge of amphibian microbiomes is important to numerous fields, including species conservation, the detection and quantification of environmental changes and stressors, and the discovery of new compounds with medical applications.

## 5. Conclusions

Amphibians are important components of most ecosystems and serve a critical role in many food webs, especially in highly diverse tropical areas. *Melanophryniscus admirabilis* is a critically endangered and microendemic species whose survival is directly related to our ability to understand its ecology, identify major anthropic impacts, and act to preserve its habitat. This work advances current knowledges of the oral microbiota of this species. Our data support the predominance of the phylum Proteobacteria in the oral microbiota of *M. admirabilis*. No significant differences among the microbial community profile from different samples were reported, suggesting that low diet diversity in this population may directly influence the bacterial composition. Oral microbiota contributed to a range of metabolic pathways, with membrane transport, amino acid metabolism, carbohydrate metabolism, replication, and repair predicted as the most prominent categories. The results of this study highlight the potential functional profiles of the xenobiotic degradation pathway in the oral microbiota of these toads. These communities might serve important roles in the health and survival of this species while also serving as an essential component of a successful conservation strategy. Therefore, our results contribute to the knowledge of ecological aspects of *M. admirabilis’s* oral microbiota, which may have important implications for the conservation and management of this critically endangered species because it occurs in a narrow range of environmental conditions and is experiencing an ongoing reduction in habitat quality.

## Figures and Tables

**Table 1:** Details of wild *Melanophryniscus admirabilis* (admirable red-belly toads) were analyzed in this study.

SAMPLE (ID)	SVL * (mm)	MASS (g)	SEX
AC421	31.51	3.1	Male
AC422	29.88	3	Male
AC423	30.63	3.2	Male
TA01 I	35.46	3.6	Female
TA02 II	33.62	3.2	Male
TA04 IV	31.50	2.7	Male
TA05	34.72	3.8	Female
TA07 VII	32.33	3.6	Male
TA10 X	33.87	4.2	Female
TA11 XI	33.67	4.1	Male
TA12 XII	35.07	3.9	Male

* SVL—Snout–vent length.

## Data Availability

The data presented in this study are openly available in European Molecular Biology Laboratory (EMBL) accession codes PRJEB33232.

## Supplementary Materials

The following are available online at https://www.mdpi.com/2076-2607/9/2/220/s1: Table S1, Percentage and average of identified phyla (>1%) among the eleven oral samples of wild *Melanophryniscus admirabilis* (admirable red-belly toads); Table S2, Percentage and average of identified order (>1%) among the eleven oral samples of wild *Melanophryniscus admirabilis* (admirable red-belly toads); Table S3, Percentage and average of identified family (>1%) among the eleven oral samples of wild *Melanophryniscus admirabilis* (admirable red-belly toads); Table S4, Relative abundance of amplicon sequence variants belonging to the persistent groups in relative KEGG Level 2 of xenobiotic/drug metabolism identified among the eleven oral samples of wild *Melanophryniscus admirabilis* (admirable red-belly toads); Figure S1, Five most abundant families presented in the eleven oral samples of wild *Melanophryniscus admirabilis* (admirable red-belly toads). Taxonomic composition of the oral microbiota among the eleven samples was compared based on the relative abundance (reads of a taxon/total reads in a sample); Figure S2., Heatmap of the significant predicted KEGG pathways from the oral bacterial community of the eleven wild *Melanophryniscus admirabilis* (admirable red-belly toads).
