# Supplementary material for: The Oral Bacterial Community in Melanophryniscus admirabilis (Admirable Red-Belly Toads): Implications for Conservation
Source: Microorganisms. 2021 Jan 22;9(2):220. doi: 10.3390/microorganisms9020220 (PMC7912307; doi:10.3390/microorganisms9020220)
Supplement: Supplementary file 1 [file microorganisms-09-00220-s001.pdf]

Supplementary Material

The oral bacterial community in *Melanophryniscus admirabilis* (admirable red-belly toads): Implications for conservation.

Michele Bertoni Mann[1], Janira Prichula[2], Ícaro Maia Santos de Castro[2], Juliana Mello Severo[2], Michelle Abadie[3], Thayná Mendes De Freitas Lima[4], Valentina Caorsi [4,5], Márcio Borges-Martins[4], Jeverson Frazzon[6], Ana Paula Guedes Frazzon[1]

Table S1. Percentage and average of identified phyla (>1%) among the eleven oral samples of wild *Melanophryniscus admirabilis* (admirable red-belly toads)

Phyla	TAO1 I	TA02 II	TA04 IV	TA05	TA07 VII	TA10 X	TA11 XI	TA12 XII	AC421	AC422	AC423	Average
**Proteobacteria**	50%	71%	62%	60%	39%	60%	66%	52%	53%	51%	22%	53%
**Firmicutes**	10%	16%	20%	17%	14%	18%	10%	16%	15%	18%	48%	18%
**Bacteroidetes**	22%	7%	9%	15%	38%	13%	12%	13%	23%	21%	17%	17%
**Actinobacteria**	6%	2%	5%	5%	5%	6%	3%	7%	5%	4%	9%	5%
**Cyanobacteria**	3%	0%	0%	0%	0%	0%	2%	8%	0%	1%	2%	2%
**Fusobacteria**	2%	1%	0%	1%	4%	1%	1%	2%	2%	4%	2%	2%
**Acidobacteria**	1%	0%	2%	0%	0%	1%	2%	1%	0%	0%	0%	1%
**Verrucomicrobia**	1%	0%	2%	0%	0%	0%	1%	0%	0%	0%	1%	0%
**Armatimonadetes**	1%	0%	0%	0%	0%	0%	1%	0%	0%	0%	0%	0%
**Spirochaetes**	0%	0%	0%	0%	0%	0%	0%	0%	1%	0%	0%	0%
**Tenericutes**	0%	0%	0%	0%	0%	0%	0%	0%	0%	0%	0%	0%
**Planctomycetes**	0%	0%	0%	0%	0%	1%	0%	0%	0%	0%	0%	0%
**Chloroflexi**	0%	0%	0%	0%	0%	0%	0%	0%	0%	0%	0%	0%
**Chlamydiae**	0%	1%	0%	0%	0%	0%	0%	0%	0%	0%	0%	0%
**Crenarchaeota**	0%	1%	0%	0%	0%	0%	0%	0%	0%	0%	0%	0%
**Nitrospirae**	0%	0%	0%	1%	0%	0%	0%	0%	0%	0%	0%	0%
**Others**	1%	0%	0%	1%	0%	0%	2%	1%	1%	1%	1%	2%
**Unknown**	1%	0%	0%	0%	0%	0%	1%	1%	0%	0%	0%	0%

Table 2. Percentage and average of identified order (>1%) among the eleven oral samples of wild *Melanophryniscus admirabilis* (admirable red-belly toads).

Order	TAO1 I	TA02 II	TA04 IV	TA05	TA07 VII	TA10 X	TA11 XI	TA12 XII	AC421	AC422	AC423	Average
**Burkholderiales**	17%	17%	22%	35%	12%	32%	33%	23%	35%	21%	9%	23%
**Bacteroidales**	19%	7%	8%	14%	24%	11%	4%	11%	21%	20%	16%	14%
**Lactobacillales**	5%	3%	4%	7%	6%	8%	4%	8%	5%	9%	31%	8%
**Clostridiales**	4%	10%	12%	10%	7%	7%	2%	4%	7%	7%	15%	8%
**Enterobacteriales**	4%	16%	12%	7%	3%	4%	10%	9%	1%	11%	1%	7%
**Pseudomonadales**	5%	22%	5%	1%	8%	4%	3%	2%	0%	1%	1%	5%
**Actinomycetales**	5%	2%	5%	4%	4%	6%	3%	6%	5%	3%	8%	5%
**Sphingomonadales**	5%	6%	10%	5%	2%	7%	7%	2%	1%	4%	2%	4%

**Neisseriales**	4%	2%	3%	4%	5%	1%	1%	4%	4%	5%	3%	3%
**Pasteurellales**	6%	3%	1%	2%	4%	1%	1%	1%	5%	5%	4%	3%
**Rhizobiales**	3%	3%	4%	4%	2%	8%	4%	3%	1%	1%	0%	3%
**Bacillales**	1%	3%	3%	0%	0%	3%	3%	2%	2%	1%	1%	2%
**Fusobacteriales**	2%	1%	0%	1%	4%	1%	1%	2%	2%	4%	2%	2%
**Others**	18%	5%	10%	5%	16%	6%	19%	20%	7%	5%	6%	11%
**Unknown**	3%	1%	1%	1%	3%	2%	6%	3%	2%	2%	1%	2%

Table 3. Percentage and average of identified family (>1%) among the eleven oral samples of wild *Melanophryniscus admirabilis* (admirable red-belly toads).

Family	TAO1 I	TA02 II	TA04 IV	TA05	TA07 VII	TA10 X	TA11 XI	TA12 XII	AC421	AC422	AC423	Average
**Burkholderiaceae**	11%	11%	15%	19%	8%	24%	27%	12%	31%	9%	7%	16%
**Prevotellaceae**	14%	6%	6%	11%	20%	6%	2%	8%	16%	16%	10%	10%
**Enterobacteriaceae**	4%	16%	12%	7%	3%	4%	10%	9%	1%	11%	1%	7%
**Streptococcaceae**	3%	1%	3%	5%	4%	3%	1%	6%	3%	7%	29%	6%
**Comamonadaceae**	2%	5%	6%	14%	3%	7%	1%	9%	4%	11%	1%	6%
**Veillonellaceae**	3%	5%	7%	9%	5%	3%	1%	2%	4%	5%	14%	5%
**Sphingomonadaceae**	5%	6%	10%	5%	2%	7%	6%	2%	1%	4%	2%	4%
**Moraxellaceae**	3%	22%	4%	1%	3%	3%	1%	1%	0%	1%	1%	3%
**Pasteurellaceae**	6%	3%	1%	2%	4%	1%	1%	4%	5%	5%	4%	3%
**Neisseriaceae**	4%	2%	3%	4%	5%	1%	1%	4%	4%	5%	3%	3%
**Bradyrhizobiaceae**	1%	3%	4%	3%	1%	6%	2%	2%	1%	1%	0%	2%
**Micrococcaceae**	2%	1%	1%	2%	3%	1%	0%	2%	2%	2%	4%	2%
**Paraprevotellaceae**	1%	1%	1%	1%	2%	3%	0%	1%	2%	2%	5%	1%
**Staphylococcaceae**	0%	3%	3%	0%	0%	2%	2%	2%	2%	0%	1%	1%
**Clostridiaceae**	1%	4%	3%	1%	1%	2%	1%	0%	2%	1%	0%	1%
**Pseudomonadaceae**	3%	1%	1%	1%	5%	0%	2%	1%	0%	0%	0%	1%
**Fusobacteriaceae**	2%	1%	0%	1%	2%	1%	0%	1%	2%	2%	1%	1%
**Porphyromonadaceae**	2%	0%	0%	2%	1%	2%	1%	1%	2%	2%	1%	1%
**Lactobacillaceae**	0%	1%	1%	2%	1%	5%	2%	1%	1%	0%	0%	1%
**Oxalobacteraceae**	3%	0%	0%	1%	1%	1%	5%	1%	0%	0%	0%	1%
**Chitinophagaceae**	2%	0%	1%	0%	1%	1%	3%	1%	2%	0%	0%	1%
**Sphingobacteriaceae**	1%	0%	0%	0%	4%	0%	4%	0%	0%	0%	0%	1%
**Caulobacteraceae**	1%	1%	4%	0%	0%	0%	0%	0%	1%	0%	0%	1%
**Corynebacteriaceae**	1%	0%	0%	0%	0%	0%	2%	3%	0%	0%	2%	1%
**Weeksellaceae**	0%	0%	0%	0%	6%	1%	0%	0%	0%	0%	0%	1%
**Carnobacteriaceae**	0%	0%	0%	0%	1%	0%	0%	1%	1%	1%	2%	1%
**Microbacteriaceae**	1%	0%	1%	0%	0%	1%	1%	0%	2%	0%	0%	1%
**Actinomycetaceae**	0%	0%	0%	1%	1%	0%	0%	1%	1%	1%	2%	1%
**Others**	15%	15%	9%	7%	10%	9%	16%	12%	6%	8%	5%	10%
**Unknown**	10%	3%	5%	1%	2%	4%	10%	13%	4%	4%	4%	5%

Table 4. Relative abundance of amplicon sequence variants belonging to the persistent groups in relative KEGG Level 2 of xenobiotic/drug metabolism identified among the eleven oral samples of wild *Melanophryniscus admirabilis* (admirable red-belly toads).

Prediction of Xenobiotic/Drug Metabolism	TAO1 I	TA02 II	TA04 IV	TA05	TA07 VII	TA10 X	TA11 XI	TA12 XII	AC421	AC422	AC423	Average	Standard Deviation
Aminobenzoate degradation	26,543	10,601	8,973	26,842	23,135	5,909	13,470	15,5851	5,663	28,225	25,530	30,067	40,670
Atrazine degradation	6,248	1,281	1,407	4,193	4,974	1,302	1,833	28,134	1,349	5,711	4,908	5,576	7,373
Benzoate degradation	46,840	23,216	16,979	52,638	45,425	9,234	22,936	271,823	10,172	51,881	48,206	54,486	70,593
Bisphenol degradation	4,201	1,449	1,135	4,777	3,102	997	2,455	27,209	1,233	4,321	4,594	5,043	7,150
Chloroalkane and chloroalkene degradation	20,729	9,522	8,404	26,265	20,413	5,494	10,887	122,095	4,809	22,317	25,829	25,160	31,591
Chlorocyclohexane and chlorobenzene degradation	5,751	2,678	2,078	7,032	6,080	866	2,713	29,006	1,290	5,908	8,003	6,491	7,493
Dioxin degradation	6,884	2,122	1,581	4,850	7,302	1,185	2,664	35,636	1,286	5,038	6,767	6,847	9,375
Drug metabolism by cytochrome P450	19,450	7,983	5,708	21,104	20,457	2,723	6,953	89,812	4,201	18,265	22,496	19,923	23,261
Ethylbenzene degradation	7,032	4,031	3,129	11,442	10,435	1,173	3,504	46,942	1,724	10,134	7,355	9,718	12,260
Fluorobenzoate degradation	5,822	2,617	1,864	5,199	6,489	818	2,541	25,821	1,188	4,651	7,490	5,864	6,661
Metabolism of xenobiotics by cytochrome P450	19,169	7,941	5,700	20,973	20,166	2,679	6,909	89,420	4,156	18,137	22,424	19,789	23,163
Naphthalene degradation	14,895	5,927	4,433	16,761	18,166	2,920	6,575	75,681	3,354	13,992	20,872	16,689	19,647
Nitrotoluene degradation	11,702	4,368	5,387	10,417	10,158	2,000	4,523	64,578	3,016	11,073	11,362	12,598	16,805
Polycyclic aromatic hydrocarbon degradation	2,991	549	223	1,301	2,102	415	1,306	10,820	687	1,940	2,851	2,290	2,845
Toluene degradation	33,755	18,146	14,374	37,192	39,358	6,942	14,039	167,743	6,884	35,684	32,328	36,950	42,999
Xylene degradation	4,036	948	484	1,664	4,612	649	1,774	22,998	723	2,819	4,018	4,066	6,155

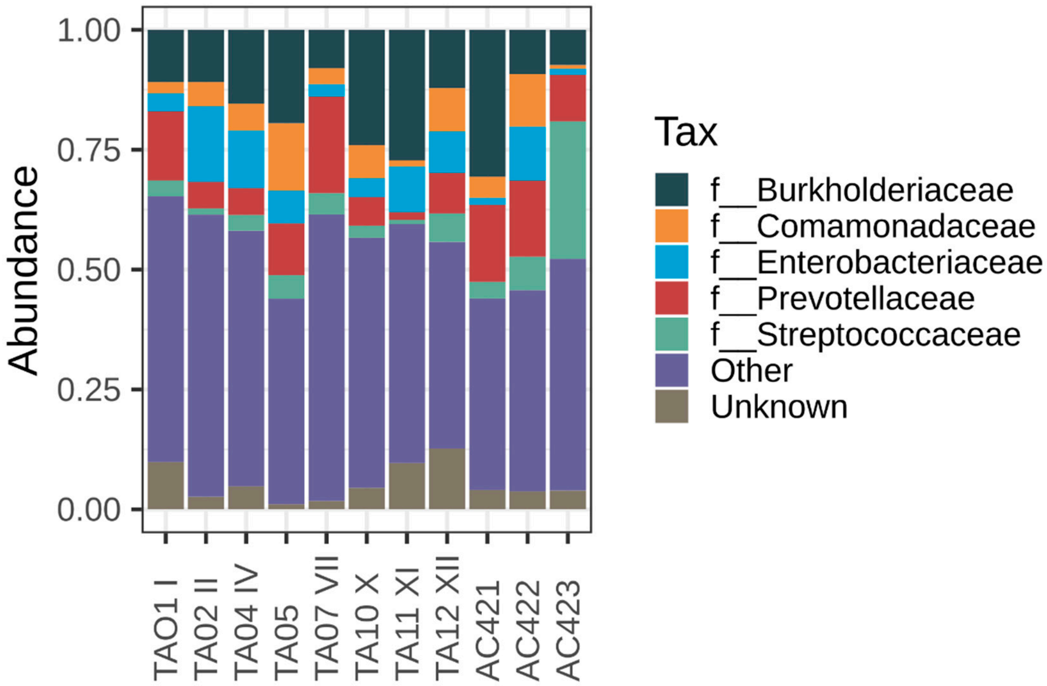

Figure 1. Five most abundant families presented in the eleven oral samples of wild *Melanophryniscus admirabilis* (admirable red-belly toads). Taxonomic composition of the oral microbiota among the eleven samples was compared based on threlative abundance (reads of a taxon/total reads in a sample).

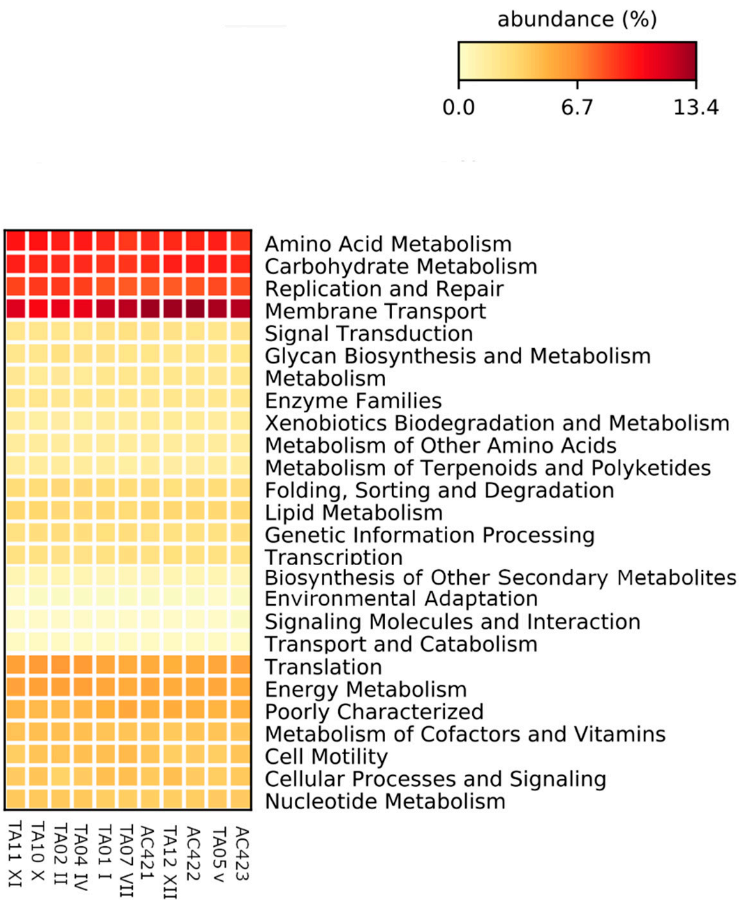

Figure 2. Heatmap of the significant predicted KEGG pathways from the oral bacterial community of the eleven wild *Melanophryniscus admirabilis* (admirable red-belly toads).
